# The safety of lookalikes: a new THC beverage enhancer and a non-THC counterpart

**DOI:** 10.1186/s42238-023-00188-7

**Published:** 2023-05-18

**Authors:** Geoffrey W. Brown, Anthony DeGelorm, Terrance J. Bellnier

**Affiliations:** 1 GPI Clinical Research, 1597 W Ridge Rd # 302, Rochester, NY 14615 USA; 2 North Carolina Poison Control, Charlotte, NC USA

**Keywords:** Cannabis product safety, Tetrahydrocannabinol, Accidental cannabis consumption, Accidental cannabis overconsumption, Accidental cannabis overdose, Toxicology, Public health

## Abstract

A new tetrahydrocannabinol (THC) beverage enhancer is available to medical and recreational cannabis consumers across the US. Beverage enhancers that do not contain THC, but instead contain flavored concentrates and/or other additives such as caffeine, are used by squirting the contents of a bottle into water, or other beverage of choice, ad libitum and can be used in a titratable manner according to the user’s preference or taste. The THC beverage enhancer described herein has an important safety feature: a mechanism that allows users to measure out a 5-mg dose of THC before they add it to their beverage. This mechanism, however, can be easily bypassed if a user attempts to use the product exactly the same way that its non-THC counterparts are used, by turning the bottle upside down and squirting the contents of the bottle into a beverage ad libitum. The THC beverage enhancer described herein would benefit from additional safety features such as a mechanism that prevents the contents of the bottle from leaving the device when turned upside down and a THC warning label.

## Background

**Fig. 1:**
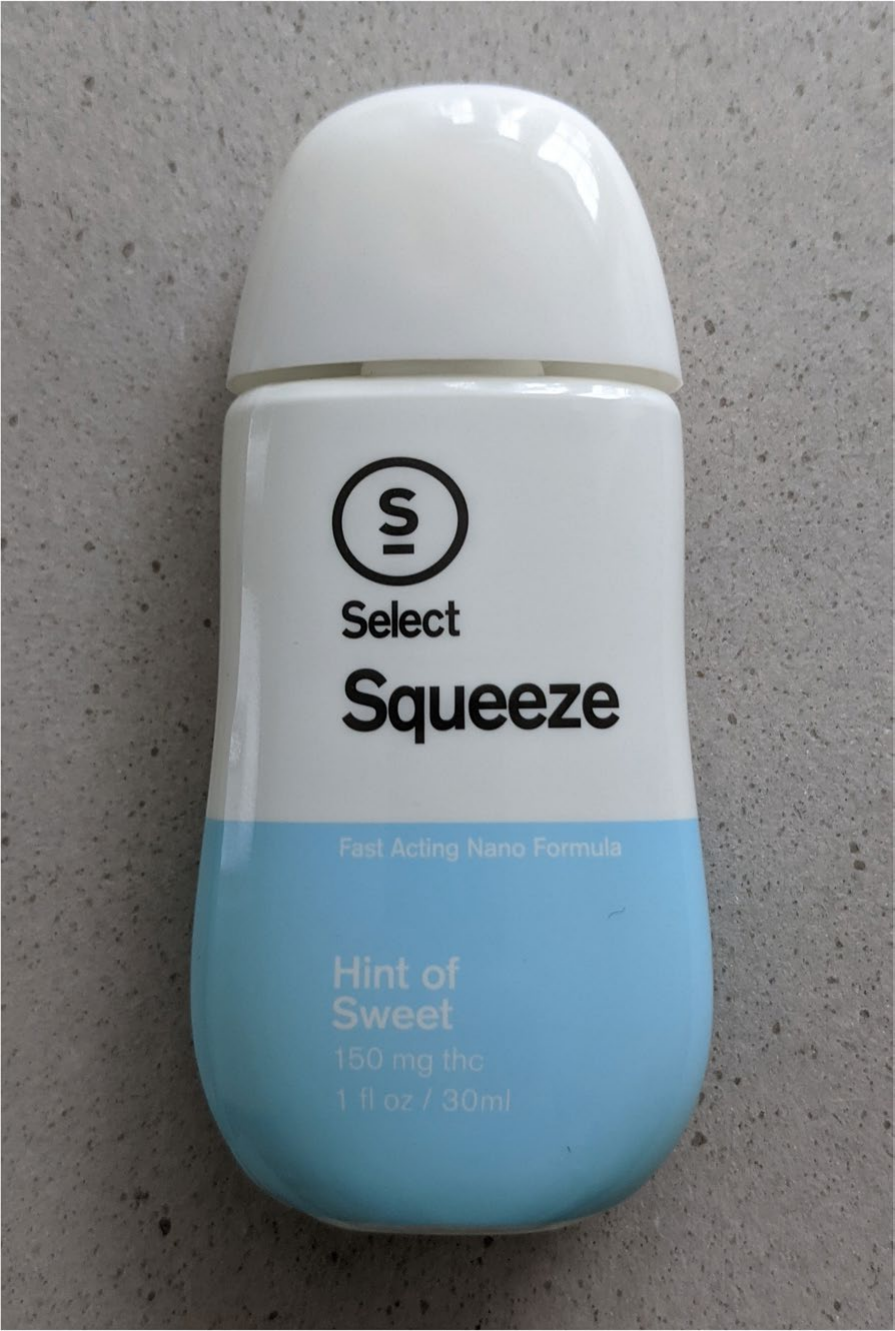
This image displays a front view of the select squeeze bottle and forward-facing product label including the name of the device, flavor of the beverage additive “Hint of Sweet”, total THC, and volume per container. The device uses a child-resistant safety cap

**Fig. 2:**
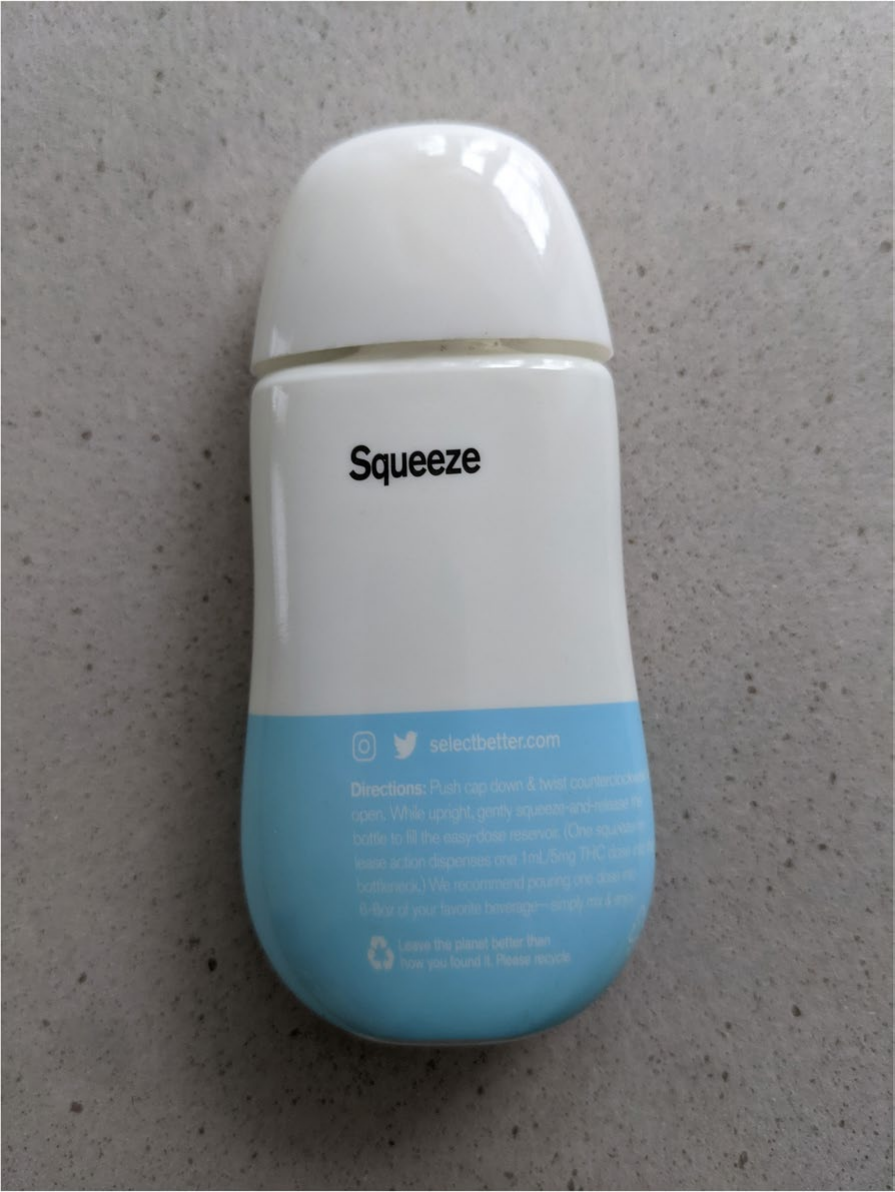
This image displays the back view of the select squeeze bottle and backward-facing product label including the select website, social media information, product instructions, and recycling insignia

**Fig. 3:**
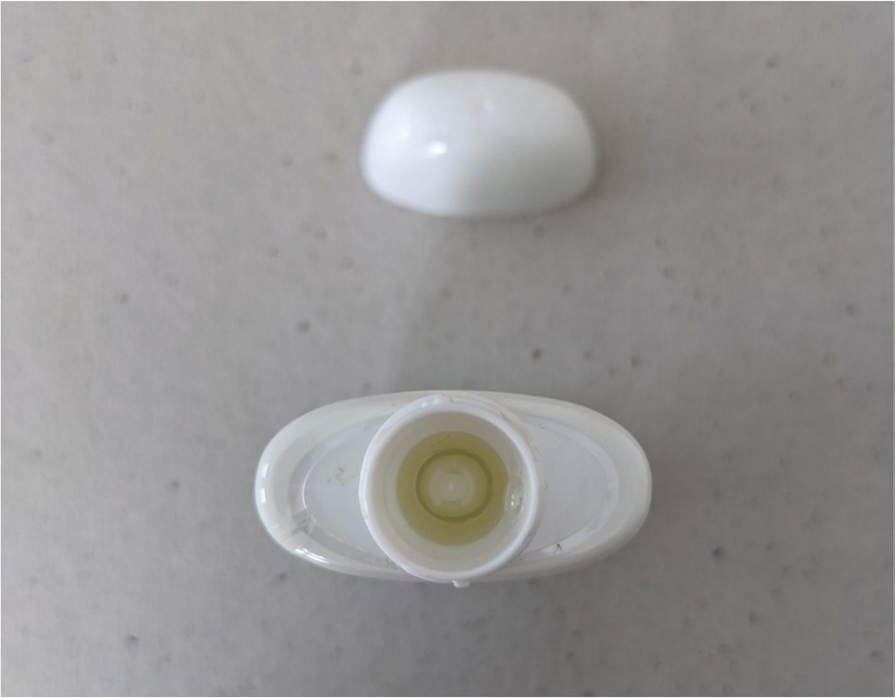
This image displays a top view of the select squeeze bottle with child-resistant safety cap removed. The dosage reservoir contained within the bottleneck of the device can be seen here as well as the overflow drain and conduit

**Fig. 4:**
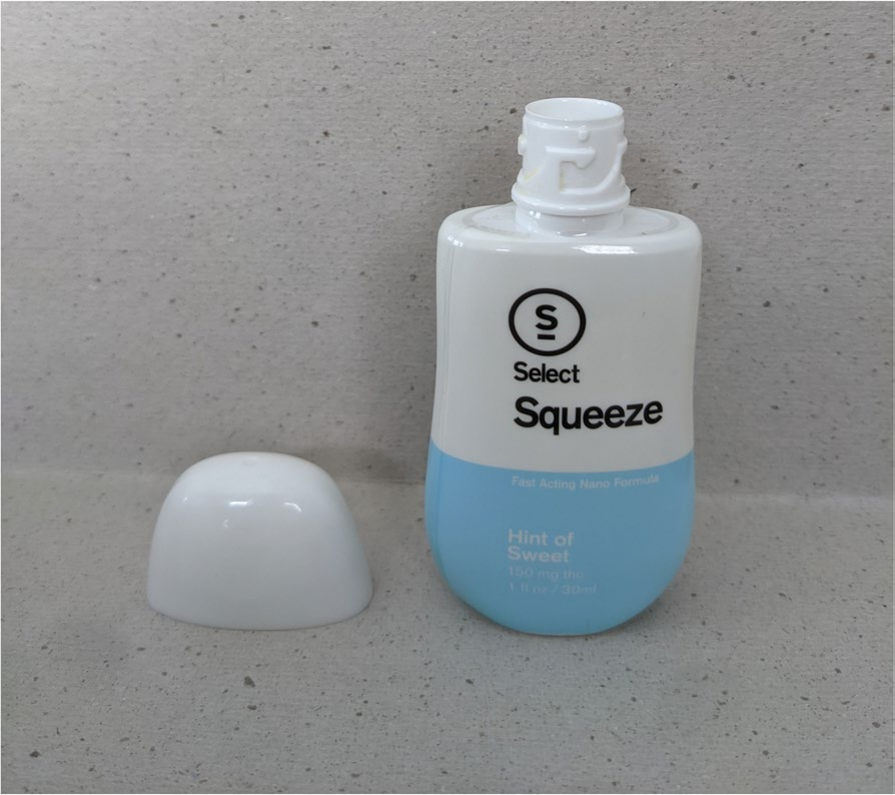
This image displays a front view of the select squeeze bottle with the child-resistant safety cap removed, revealing the bottleneck of the device which contains the dosage reservoir

In March of 2021, cannabis behemoth Curaleaf announced the launch of Select Squeeze (SQZ), a fast-acting, tetrahydrocannabinol (THC)-containing, liquid beverage enhancer (Curaleaf Holdings, Inc. 2021). With a market capitalization of more than $6 billion, Curaleaf is among the largest cannabis companies in the US operating in 23 states and Europe with an addressable population of more than 400 million adults. The debut of SQZ across the US is likely the widest national cannabis product launch in industry history. SQZ contains an aqueous solution composed of cannabis extract with added water, preservatives, flavors, and coloring agents that improve product appeal, stability, and shelf life (Squeeze and [Package Insert] 2020). SQZ is formulated using nano-emulsification technology that disperses THC molecules and other cannabinoids in colloidal nanoparticles enabling enhanced water solubility, quicker onset of action, and increased bioavailability (Curaleaf Holdings, Inc. 2021; Squeeze and [Package Insert] 2020). Squeeze is provided in a specialized, easy dose, fill-and-pour device. Photographs of the device are provided in Figs. 1, 2, 3, and 4.

The directions for use written on the SQZ product label, shown in Fig. 2, are as follows: “Push cap down & twist counterclockwise to open. While upright, gently squeeze-and-release the bottle to fill the easy-dose reservoir. (One squeeze release action dispenses a 1 mL/5 mg THC dose into the bottleneck). We recommend pouring one dose into 6-8 oz of your favorite beverage –simply mix & enjoy.”

**Fig. 5:**
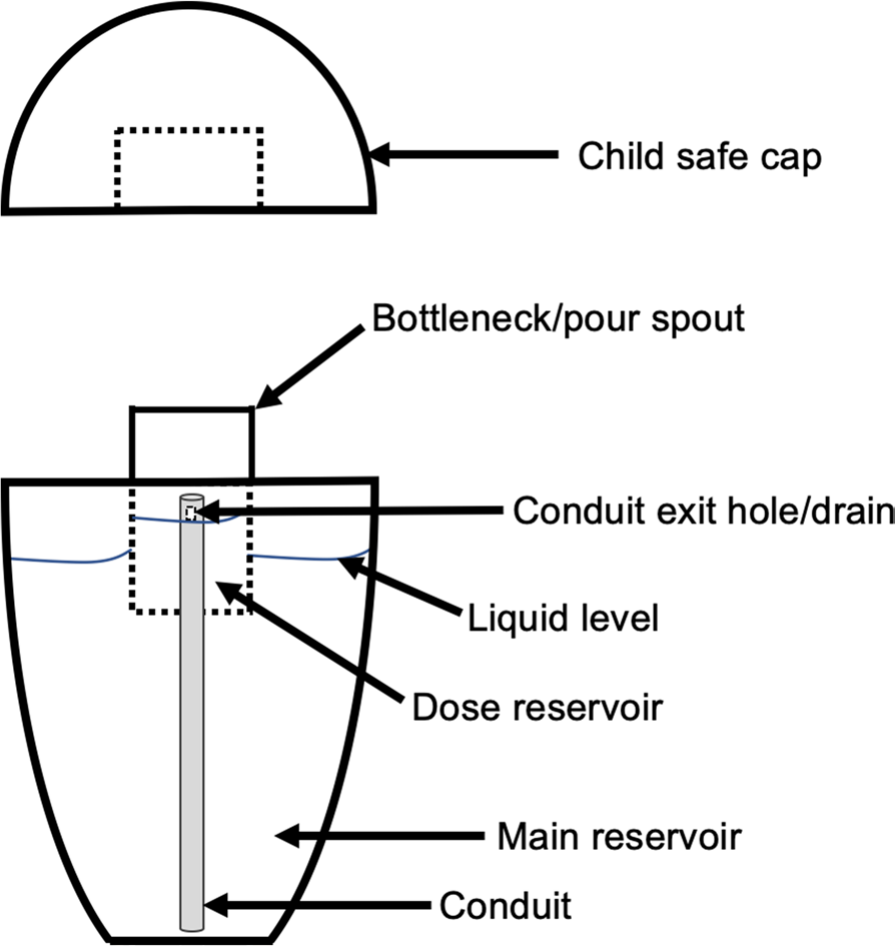
This diagram illustrates the details of the select squeeze device and how the easy dose fill and pour mechanism works. A 1-mL dosage reservoir is contained within the bottleneck of the device. When the safety cap is removed and while the device is being held upward, the user squeezes the outside of the bottle which increases pressure inside the main reservoir. This forces the liquid beverage enhancer up the conduit into the dosage reservoir. The conduit has drainage holes placed at a precise height as to serve as an overflow drain. This ensures that no more than 1 mL of liquid beverage enhancer or 5 mg of THC is contained within the dosage reservoir

Figure 5 shows a schematic diagram of the device. The easy-dose reservoir is filled by a conduit that extends from the bottom of the main reservoir of the device into the dose reservoir located in the device’s bottleneck. When a user squeezes the outside of the bottle, pressure inside the main reservoir is increased forcing liquid up the conduit and into the dose reservoir. Holes at the top of the conduit allow liquid to enter the dose reservoir and also act as an overflow drain. The precise placement of the holes at the proper height on the conduit ensures that the volume of liquid inside the reservoir never exceeds 1 mL because any excess volume will automatically drain back into the main reservoir.

## Look-alike products and potential for misuse

**Fig. 6:**
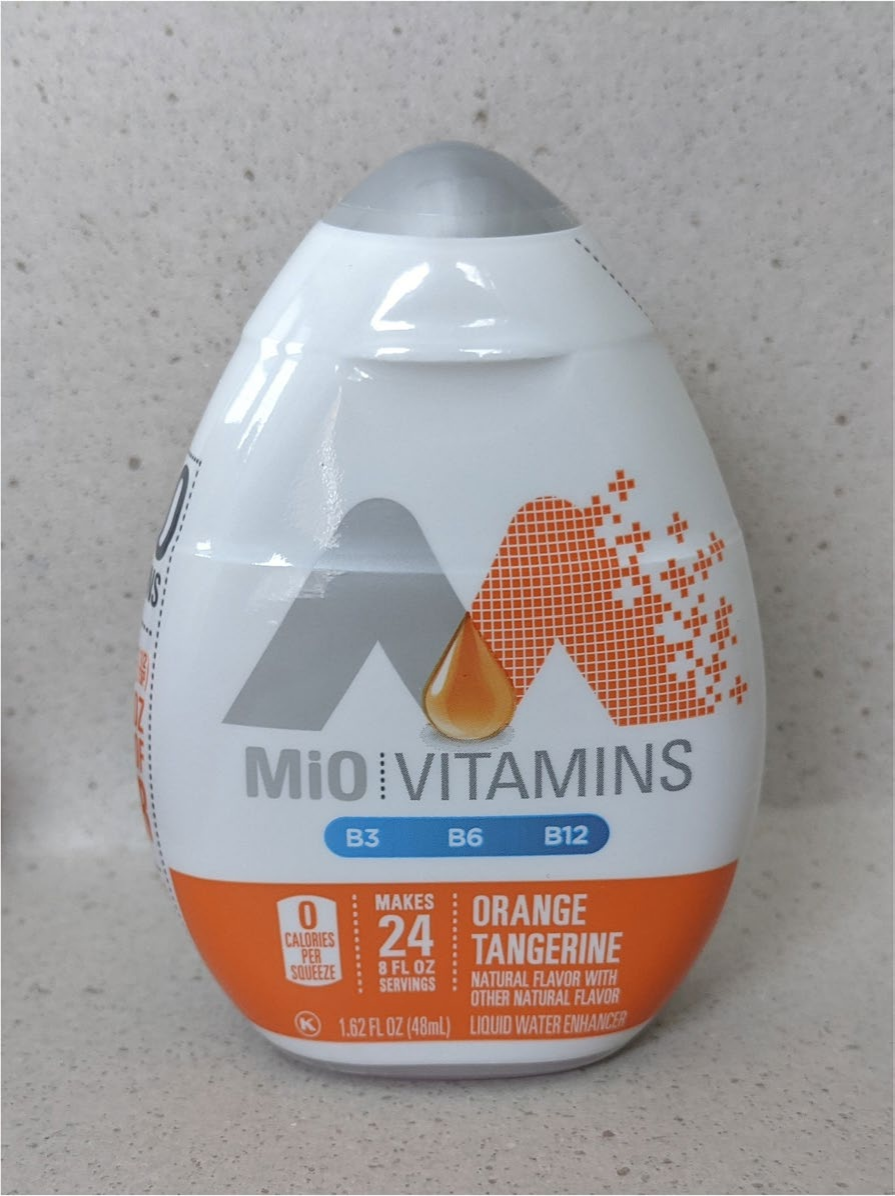
This image displays a front view of a bottle of MiO®, a popular beverage enhancer product that commonly includes food-grade ingredients such as vitamins, natural flavors, and/or caffeine. The image is provided to show the potential resemblance and similarities to the squeeze device

The SQZ device bears a close resemblance to other commercially available liquid beverage-enhancing products that do not contain THC. These non-THC-containing beverage-enhancers may contain flavoring agents, caffeine, or other dietary ingredients and include brands such as MiO®. A photograph of a bottle of MiO® is shown in Fig. 6. Liquid beverage-enhancers such as MiO® are used in a similar, but distinctly different manner than SQZ. Users may add MiO® to water, or other beverages, ad libitum by inverting the bottle and squeezing it directly into the beverage they intend to consume. This allows for an imprecise amount of flavored concentrate to be added in a titratable manner.

The SQZ device can mistakenly be used this same way. The easy-dose reservoir, which allows users to measure out 1 mL/5 mg THC doses, may be easily bypassed by inverting the device and squeezing it directly into a beverage. Doing so enables liquid to exit the device directly from the conduit without proper measurement. Using the product in this manner results in the expulsion of an unknown volume of liquid and dose of THC directly into a beverage the user intends to consume. Consequences of this can include additional side effects, adverse events, and overdose.

## Discussion

The global market for cannabis beverages is projected to exceed $19 billion by 2028 (Fortune Business Insights 2022). SQZ is available to more than 94 million adults above the age of 21 which includes medical cannabis patients and recreational consumers (Curaleaf Holdings, Inc. 2021). Medical cannabis patients tend to be older and in poorer health compared to recreational cannabis consumers (Lin et al. 2016). Older individuals may be more sensitive to neuropsychiatric side effects, somnolence, dizziness, and hypotension due to THC. Therefore, medical cannabis patients may be at an increased risk of harm from overconsumption of THC.

Figures 1, 2, 3, and 4 and Fig. 6 display photographs of SQZ and MiO® respectively. It is clear that SQZ bears somewhat of a close resemblance to other non-THC liquid beverage-enhancing products such as MiO®. When the SQZ product is inverted and squeezed directly into a beverage, as other non-THC liquid beverage-enhancers are used, an indeterminate volume of liquid is expelled from the device resulting in an unknown dose of THC added to the user’s beverage. If SQZ is used improperly in this fashion, it may lead to accidental overconsumption by exposing the user to higher than intended doses of THC.

It is important to mention that SQZ does utilize a child-resistant safety cap as is required by State-law. Due to the incredible growth of the cannabis industry and changing policy throughout the US, recreational cannabis products are becoming more accessible not only to adults, but also to children. Cannabis product exposures in children have grown in recent years and have led to an increase in calls to poison control centers and visits to healthcare facilities (Whitehill et al. 2021). In general, the use of standard child-resistant safety caps on medications has been shown to decrease pediatric medication exposures over the years, but research suggests that 45–55% of accidental medication exposures in children still involve child-resistant packaging (MacKay and Samuel 2018). Products like SQZ would do well to employ even more secure closure systems in order to further prevent pediatric exposures.

The SQZ device would also benefit from other additional safety features. First and foremost, a mechanism that would prevent the solution from being expelled from the device when it is held upside down would drastically improve product safety. Further differentiation of the SQZ bottle would also help distinguish it from other non-THC lookalike products. Placement of an explicit warning message on the product label notifying users that “this product contains THC” would also be beneficial. While this warning message was likely included on the product’s original packaging, inclusion of the warning message on the final product itself is also important given that users may discard the original packaging after using the product for the first time.

## Conclusion

As technology advances and the commercialization of cannabis continues, product safety remains an important aspect of product development. SQZ is a novel, dose-metered product, with safety features intended to improve consumer safety when used correctly, but the product has important shortcomings that may result in overconsumption and overdose if used incorrectly or without proper guidance.

Overconsumption and overdose of cannabis and THC have been associated with significant healthcare utilization and costs (Shen et al. 2019). As the US healthcare system continues to face significant hardship following the COVID-19 pandemic, it is more important than ever that we avoid unwanted strain on our health systems such as emergency room visits due to cannabis overconsumption and intoxication. Federal regulation of cannabis will improve the safety of cannabis products and protect the public health of Americans.

## Data Availability

All information provided is publicly available.

## Abbreviations

THC: Tetrahydrocannabinol
SQZ: Select Squeeze
US: United States
